# Effects of the Partial Substitution of Corn with Wheat or Barley on the Growth Performance, Blood Antioxidant Capacity, Intestinal Health and Fecal Microbial Composition of Growing Pigs

**DOI:** 10.3390/antiox11081614

**Published:** 2022-08-20

**Authors:** Xiaokang Ma, Zhiqing Li, Yuebo Zhang

**Affiliations:** College of Animal Science and Technology, Hunan Agricultural University, Changsha 410128, China

**Keywords:** pigs, wheat, barley, blood antioxidant capacity, fecal microflora

## Abstract

This experiment aimed to investigate the effects of wheat and barley substitution for corn on growth performance, blood antioxidant capacity, intestinal health and fecal microbial composition in growing pigs. Eighteen healthy “Duroc × Landrace × Yorkshire” pigs (50 ± 0.85 kg) were randomly divided into three groups with six replicates and one pig per replicate. The three treatment groups were fed the basal diet (CON) based on corn and soybean meal, respectively, and the experimental group diet was partially replaced by wheat (WH) and barley (BL), respectively. The nutritional levels of the three treatments were the same. The experiment lasted 28 days. Wheat and barley partially replacing corn had no significant effects on growing pigs’ growth performance, blood antioxidant capacity and nutrient digestibility (*p* > 0.05). Compared with CON and BL, WH significantly increased the duodenal villus height and villus height/crypt depth ratio of growing pigs (*p* < 0.05). Compared with CON, WH and BL significantly increased the contents of butyric acid, propionic acid and total volatile fatty acid (VFA) in the cecum and colonic digesta of growing pigs (*p* < 0.05). In addition, the abundance of *Turicibacter*, *Escherichia-Shigella* and other harmful bacteria in barley and wheat diet groups were significantly decreased at the genus level (*p* < 0.05). The abundance of *Bifidobacterium*, *Lactobacillus*, *Prevotella and Roseburia* increased significantly (*p* < 0.05). In conclusion, barley and wheat partially replacing corn as energy feedstuffs does not affect the growth performance of pigs but can regulate intestinal flora and promote intestinal health.

## 1. Introduction

In recent years, with the rapid development of pig breeding, the shortage of corn resources and the rising price of corn have become the primary problem to be solved in animal husbandry, and the search for feed materials to replace corn has become a research focus. Wheat and barley have been widely used as energy feed in pig breeding. In Europe, wheat has been used as the primary energy feed in animal husbandry, so wheat and barley have become the primary choice to replace corn. Many studies show that reasonable use of wheat resources in pig feed can partially or entirely replace corn and a superior feeding effect and greater economic benefit could be achieved. Barreram et al. [1] reported that adding xylanase to a growing pig diet supplemented with wheat increased average daily gain and feed conversion efficiency. Some nutrients of barley and wheat were higher than those of corn, but the non-starch polysaccharide content of the barley and wheat were higher, affecting the utilization efficiency of barley and wheat in pig feed. In addition, due to the presence of phytase in wheat raw materials, although the digestibility of the phosphorus in barley and wheat is higher than that of corn, the degradation efficiency of phytate phosphorus is lower. Cromwell et al. [2] showed that barley and wheat could replace corn from the weaning to fattening period as long as the balance of protein or amino acids in the pig diet was maintained. Therefore, the utilization efficiency of barley and wheat in the feed industry can be increased amid the current situation of high corn prices. This study was conducted to study the effects of using barley and wheat instead of corn on the growth performance, blood antioxidant capacity, nutrient digestibility, intestinal health and fecal microbial composition of growing pigs.

## 2. Materials and Methods

### 2.1. Animal Treatment and Experimental Design

Eighteen healthy “Duroc × Landrace × Yorkshire” crossbred growing pigs with similar initial body weight (50.00 ± 0.85 kg) were randomly divided into three treatment groups with six replicates (sex balanced) per treatment group and one pig per replicate. The pigs in the control group, WH group and BL group had different diets. The basal diet (CON) was based on corn and soybean meal, and pigs in the experimental treatments were fed with wheat (WH) and barley (BL) instead of corn, respectively. In addition, phytase and xylanase were supplemented in the diets of experimental groups at 500 FTU/kg and 3000 U/kg, respectively. The diets were formulated according to the nutritional requirements of growing pigs at the 50 to 80 kg stage of NRC (2012) [3]. The contents of digestible energy, crude protein, dry matter and five major essential amino acids (lysine, methionine, threonine, tryptophan and valine) in the three dietary formulas were the same. The dietary composition and nutrient levels were shown in Table 1.

The protocol of this study was approved by the Institution Animal Care and Use Committee of College of Animal Science and Technology (ACC2022075), Hunan Agricultural University (Changsha, China). The animal feeding experiment was conducted at the Liuyang Animal Testing Base in Hunan Province. The experimental period was 28 days and each piglet was free to drink and eat. During the experiment, the piglets were immunized, and the feces of the pens were cleaned every day. The ambient temperature in the house was automatically regulated by the thermostat, and the window was opened for ventilation at regular times. During the experimental period, the average daily feed intake (ADFI) was manually recorded, and the health status of pigs was observed. On the 28th day of the experiment, all the pigs fasted for 12 h. Ten mL of blood was collected from the jugular vein of the pigs to separate the serum, and then the pigs were slaughtered, and samples were collected. The intestines were quickly separated, and about 10 g of samples of chyme from in the cecum and colon were collected, mixed and stored in the −80 ℃ refrigerator for volatile fatty acid analysis. Then, intestinal histomorphological samples were taken, and about 10 g fresh feces samples were taken and stored in a −80 ℃ refrigerator for subsequent microbial composition analysis. Feed samples and fecal samples were collected at the beginning and end of the experiment for the determination of apparent nutrient digestibility. After collection, all feed and fecal samples were stored freshly at −20 °C until the analysis.

### 2.2. Direction Indicators

#### 2.2.1. Growth Performance

Average daily gain (ADG) and feed to gain ratio (F/G) were calculated based on body weight (BW) and average daily feed intake (ADFI), measured weekly.

#### 2.2.2. Blood Antioxidant Capacity

According to the manufacturer’s instructions (Jiangsu Meimian industrial Co., Ltd., Yancheng, China), the contents of the total antioxidant capacity (T-AOC), superoxide dismutase (SOD), glutathione peroxidase (GSH-Px) and malondialdehyde (MDA) in the serum of growing pigs were measured with a spectrophotometer (Cold Light sfz160017568, Shanghai, China).

#### 2.2.3. Morphology of the Small Intestine

At the end of the experiment, all the pigs were slaughtered, and the duodenum, jejunum and ileum of the pigs were collected for the production of small intestine slices [4]. The intestinal villi height and recess depth of pigs were measured by optical microscope (IPP; American media cybernetics company, Washington, DC, USA).

#### 2.2.4. Volatile Fatty Acid Analysis

According to the method of Yu et al. [5], the content of volatile fatty acids in cecal chyme was determined by gas chromatography (7890B, Agilent Technologies Inc., Santa Clara, CA, USA). Short chain fatty acid (SCFA) content was calculated by a standard curve using a standard external method.

#### 2.2.5. Nutrient Digestibility

During this experiment, 1 kg of feed samples were collected weekly. The dry matter (DM), crude protein (CP), gross energy (GE) and organic matter (OM) in feed and fecal samples were measured according to AOAC (2012).

The apparent total tract digestibility (ATTD) was calculated as follows:

ATTD nutrient = 1 − (Cr_diet_ · Nutrient_feces_)/(Cr_feces_ · Nutrient_diet_). (1)

#### 2.2.6. Microbiota Analysis by 16S RNA

The intestinal contents were collected and put in the EP tube after high-temperature sterilization, and then extract the total DNA from the pig fecal samples using the Qia amp fast DNA Kit (Qiagen, Germany). After completing the DNA extraction, use 1% agarose gel electrophoresis to detect the quality of the extracted DNA. The ABI gene amp r9700 PCR thermal cycler (ABI, Los Angeles, CA, USA) was used for amplification. Then, the PCR products were extracted, purified and quantified. Paired-end sequencing was performed on the Illumina miseq pe300 platform/novaseq PE 250 platform (Illumina, San Diego, CA, USA). According to previous studies [6,7], the original 16S rRNA gene sequencing reads were demultiplexed, quality filtered and merged. The microbial communities were calculated using Alpha Diversity, (1) Chao1: community richness; (2) Shannon: the index of community diversity. The analysis of similarity (anosim) of the vegan package in R (version 3.1.2) was used to test the community similarity; the PAM package was used for the nearest shrenken centroid (NSC) analysis to screen core OTUs of different intestinal segments. Linear discriminant analysis (LDA) was carried out using the online tool (http://huttenhower.sph.harvard.edu/galaxy/ (accessed on 16 May 2022) to find out the strains that have a significant difference in the typing of different intestinal flora. The Kruskal-Wallis test was used to analyze the relative abundance of microorganisms in different intestinal segments and different treatments at the taxonomic level of phyla, family and genus, and Tukey’s HSD (Tukey’s Honey Significant Difference) was used for multiple comparisons. Benjamin Hochberg’s FDR method was used to correct the *p*-value. The correlation analysis of microbial abundance in each intestinal segment was calculated in JMP 10.0 by the Spearman method.

### 2.3. Statistical Analysis

All data were analyzed using SAS (version 9.2; SAS Inst. Inc., Cary, NC, USA). All data were analyzed using a student’s *t*-test for unpaired data with each pen or pig as an experimental unit. The results were presented as the mean and standard error of means (SEM). The significant difference was declared at *p* < 0.05.

## 3. Results

As shown in Table 2, the partial substitution of corn by wheat or barley had no significant effect on the final weight, average daily feed intake, average daily gain and feed over gain ratio of growing pigs (*p* > 0.05).

It can be seen from Table 3 that compared with CON, WH and BL had no significant effect on the blood antioxidant capacity of growing pigs (*p* > 0.05).

According to Table 4, compared with CON, WH and BL had no significant effect on the growing pigs’ total intestinal nutrient apparent digestibility (*p* > 0.05).

As can be seen from Table 5, Compared with CON and BL, WH significantly increased the villus height and the ratio of villus height to crypt depth in the duodenum of growing pigs (*p* < 0.05).

As shown in Table 6, compared with CON and WH, BL significantly increased the contents of acetic acid and total VFA in the cecal digesta of growing pigs (*p* < 0.05). Compared with CON, WH and BL can significantly increase the content of butyric acid in the cecal digesta of growing pigs (*p* < 0.05). In addition, compared with CON and WH, BL significantly increased the contents of propionic acid and total VFA in the colonic digesta of growing pigs (*p* < 0.05).

**Figure 1:**
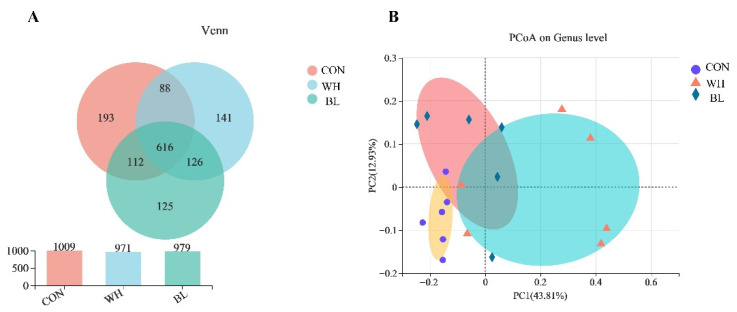
(**A**) Effects of the partial substitution of corn by wheat or barley on the OTU of fecal microflora in growing pigs. (**B**) Principal coordinate analysis (PCoA) of the fecal microbial composition of growing pigs (based on the Bray-Curtis distance). The individual pig was regarded as the experimental unit (n = 6). CON, basal diet group; WH, wheat diet group; BL, barley diet group.

The Venn diagram showed that the OTUs in the fecal samples of growing pigs in CON, WH and BL were 1009, 971 and 979, respectively, with a total of 688 OTUs in the three groups (Figure 1A). Principal coordinate analysis (PCoA) was used to characterize the β diversity of bacterial communities in the fecal samples of growing pigs in CON, WH and BL groups (Figure 1B). PCoA with Bray-Curtis distance showed significant segregation in all three groups, suggesting that the microbial community composition of the three groups was significantly different. 

**Figure 2:**
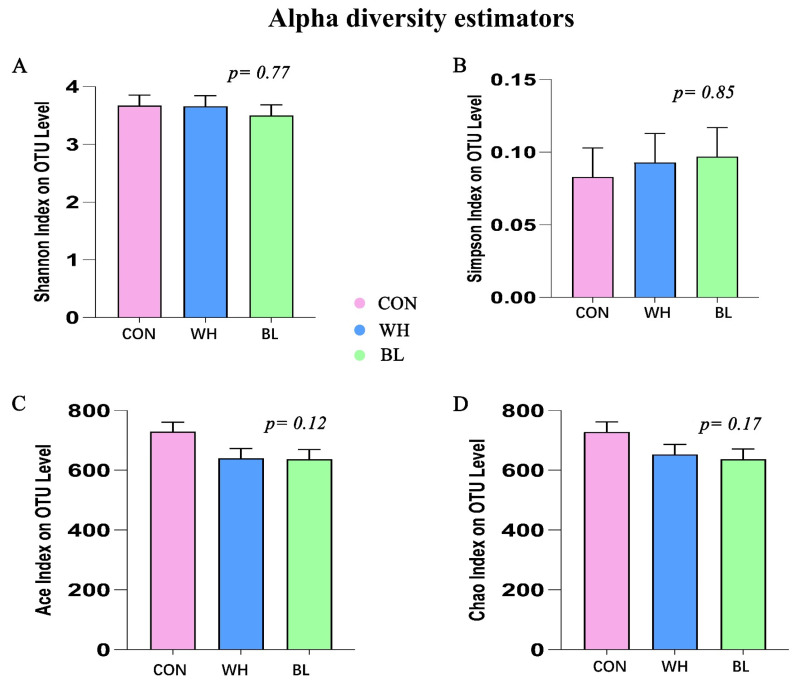
Effects of the partial substitution of corn by wheat or barley on the alpha diversity of bacterial flora in the feces of growing pigs. (**A**) Shannon index, (**B**) Simpson index, (**C**) Ace index, (**D**) Chao index. The individual pig was regarded as the experimental unit (n = 6). CON, basal diet group; WH, wheat diet group; BL, barley diet group.

There were no significant differences in the Shannon index, Simpson index, Ace index and Chao index among the three groups (*p* > 0.05, Figure 2).

**Figure 3:**
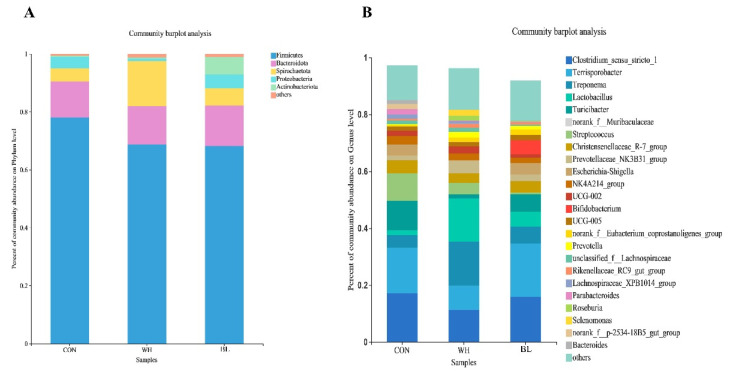
Relative abundance of fecal bacteria based on the phylum (**A**) and genus (**B**) level. Different color areas in the figure indicate the proportions of different species. Among them, species with an abundance less than 1% are combined as other. The individual pig was regarded as the experimental unit (n = 6). CON, basal diet group; WH, wheat diet group; BL, barley diet group.

As shown in Figure 3, at the phylum level, a total of five phyla were detected in three groups of samples.

Different colors in the figure represent different bacterial communities. The higher the column, the higher the relative abundance of microorganisms. The relative abundance and proportion of each group at the phylum level could be intuitively seen from the species annotation results. Seven dominant bacteria were identified at the phylum level of pig feces. *Firmicutes, Bacteroidota* and *Spirochaetota* were the dominant phyla, accounting for 78.06, 12.39 and 4.53%, respectively, in CON treatment, accounting for 68.75, 17.18 and 15.60%, respectively, in WH treatment and accounting for 68.19, 13.91 and 5.98%, respectively, in BL treatment (Figure 3A). At the genus level, a total of 24 genera were detected in the three groups. Different colors in the figure represent different bacterial communities. The higher the column, the larger the proportion of the sample and the higher the relative abundance. The relative abundance and proportion of each group at the genus level could be seen from the annotation results. The dominant bacteria in CON treatment were *Clostridium_sensu_stricto_1*, *Terrisporobacter* and *Turicibacter*. The proportions were 17.19, 16.05 and 10.44%, respectively. The dominant bacteria in WH treatment were *Lactobacillus, Treponema* and *Clostridium_sensu_stricto_1*, accounting for 15.24, 15.47 and 11.34%, respectively. The dominant genera in BL treatment were *Terrisporobacter, Clostridium_sensu_stricto_1* and *norank_f_Muribaculaceae*, accounting for 18.81, 15.98 and 8.06%, respectively (Figure 3B).

**Figure 4:**
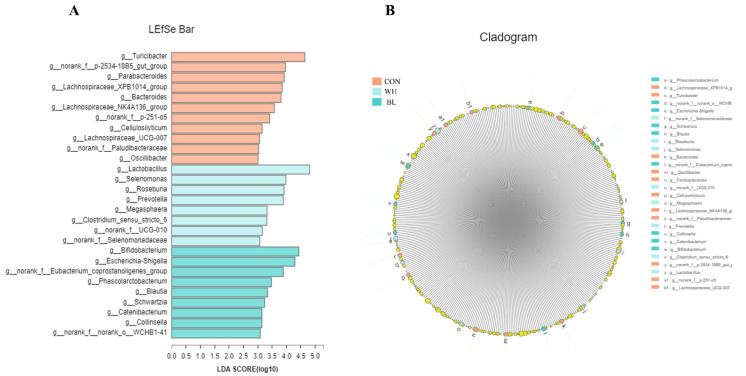
Identification of the most differentially abundant genera in fecal. The plot is generated via linear discriminant analysis effect size (LEfSe) analysis with a CSS-normalized OTU table, which displays taxa with latent dirichlet allocation (LDA) scores above 2 and *p*-values below 0.05. (**A**) LEfSe Bar, (**B**) Cladogram. The individual pig was regarded as the experimental unit (n = 6). CON, basal diet group; WH, wheat diet group; BL, barley diet group.

Lefse analysis was used to analyze and identify bacteria with significant differences at the gate level between the three treatments (Figure 4, LDA = 2). In CON, WH and BL treatment groups, bacteria in 11, 8 and 9 were significantly enriched.

**Figure 5:**
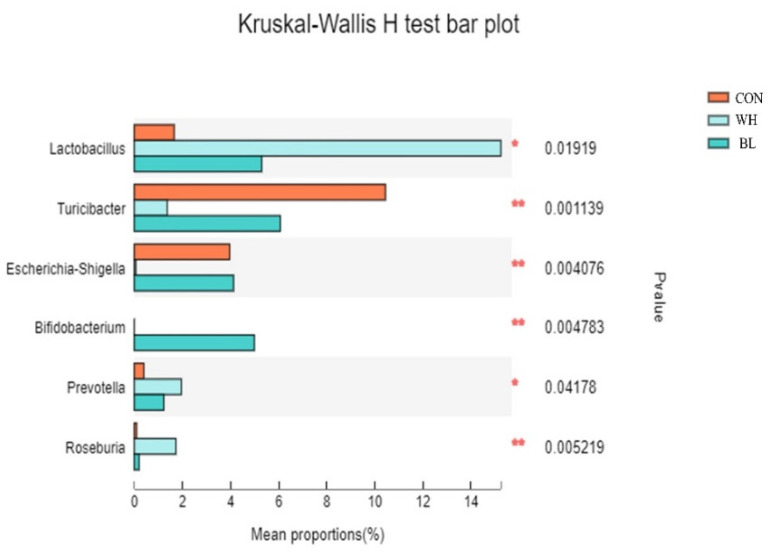
The fecal samples of growing pigs fed three different diets were tested for significant differences between dominant bacteria genera. The Kruskal–Wallis test, followed by Tukey’s test, was used to evaluate the statistical significance. Asterisks express statistical differences between different groups: * 0.01 < *p* ≤ 0.05. ** *p* ≤ 0.01. The individual pig was regarded as the experimental unit (n = 6). CON, basal diet group; WH, wheat diet group; BL, barley diet group.

As can be seen from Figure 5, the results showed that there were significant differences in the composition of genus level species in the feces of the three groups.

At the genus level, *Turicibacter*, *Escherichia*-*Shigella*, *Bifidobacterium*, *Lactobacillus*, *Prevotella* and *Roseburia* bacteria with relative abundance in the CON, WH, BL demonstrate significant differences between the three treatment groups (*p* < 0.05).

## 4. Discussion

Feeding cereal diets rich in water-soluble non-starch polysaccharides, such as wheat, barley and rice, increases the intestinal chyme viscosity and reduces the contact area between chyme and digestive enzymes in the gastrointestinal tract of animals. However, the utilization efficiency of some nutrients can be improved along with the appropriate supplementation of enzyme preparations. Nasir et al. [8] found that barley partially replacing corn had no negative impact on the growth performance of piglets. Bruneau [9] and Mavromichalis et al. [10] also found that wheat has high palatability to pigs. When phytase is added to the wheat diet, more energy and protein can be released in wheat under the action of phytase. This allows growing pigs fed with barley and wheat to achieve the same growth performance as those fed with corn. This study also showed that barley and wheat diets had no significant effects on the average daily gain, average daily feed intake and feed conversion rate of growing pigs. Therefore, under the condition of meeting the amino acid and protein requirements of growing-finishing pigs, adding an appropriate level of barley or wheat to the diet does not affect the growth performance of pigs.

Phytase and xylanase are typically added to wheat-based diets because combining phytase and xylanase significantly improves pig growth performance and nutrient digestibility [11]. It has been reported that the digestibility of crude protein and amino acids is higher in wheat-type diets. However, the digestibility of nitrogen and dry matter is no different from that of corn [12]. In this study, dietary treatment did not significantly affect the apparent digestibility of dry matter, crude protein and the gross energy of growing pigs. The results of this experiment are inconsistent with those of previous studies, which may be caused by the difference between barley and wheat feed materials selected in this experiment.

The integrity of intestinal morphology is crucial for the growth of pigs, and the complete intestinal morphology is more conducive to the digestion and absorption of nutrients by pigs [13]. This study showed that the partial substitution of corn by wheat and barley had no significant effect on the integrity of the intestinal morphology of growing pigs. Willamil et al. [14] also found that compared with the corn-soybean meal diet, piglets fed barley and wheat diets supplemented with 0.01% complex enzymes (xylanase and β-glucanase) tended to have a higher average daily gain, healthier intestinal morphology and better nutrient utilization efficiency.

Microorganisms ferment in the cecum and colon of pigs and can produce a variety of volatile fatty acids, which plays an important role in protecting the intestinal health of pigs [15]. A resistant starch diet not only significantly increased the concentration of short-chain fatty acids in the cecum and colon, but also regulated the composition of intestinal microflora and affected the expression of short-chain fatty acid-related genes [16]. Diebold et al. [17] found that xylanase supplementation in wheat diets increased the amounts of acetic acid and propionic acid in the intestines of piglets. Yin et al. [18] also found similar results. Adding xylanase to the wheat-based diet of finishing pigs increased the content of intestinal volatile fatty acids. This study also showed that phytase and xylanase supplementation in barley and wheat diets produced more volatile fatty acids in the cecum and colon of pigs, which is highly significant for the improvement of the intestinal health of pigs.

The microbiome in the gut plays a vital role in maintaining animal nutrition and immunity. Microorganisms can help animals digest and utilize nutrients in the diet, assist animal metabolism, provide nutrients for intestinal epithelial cells and help hosts resist the invasion of pathogenic bacteria by strengthening the immune and disease resistance function of intestinal cells [19]. There is a wide variety of microorganisms in the gastrointestinal tract of monogastric animals, including bacteria, fungi, viruses and parasites, all of which play a critical role in maintaining the host’s normal metabolism and immune health [20]. This study found that the microbial community in pig feces had changed. This may be due to barley and wheat containing more crude fiber; crude fiber could be directly used as microbial energy material. Therefore, different fiber types and contents will change the species and relative abundance of microorganisms [21]. In this experiment, through the test and analysis of the significance of differences between the microbiome, it was found that at the genus level, the abundance of harmful bacteria such as *Turicibacter* and *Escherichia-Shigella* in barley and wheat diet groups was significantly decreased, while the abundance of beneficial bacteria such as *Bifidobacterium*, *Lactobacillus*, *Prevotella* and *Roseburia* was significantly increased, compared to the corn group. Drew et al. [22] also showed that compared with the corn-fed diet, the relative abundance of lactobacillus in the chyme of piglets fed with wheat feed increased, while the relative abundance of *Escherichia coli* decreased, and the abundance of *Bifidobacteria* in cecum also significantly increased. *Rosebaria* is a bacterium that produces butyric acid. Butyric acid is the primary energy substance for colon cells, providing energy for colon epithelial cells and preventing colon inflammation [23]. Donohoe et al. [24] also found that butyric acid functions as an amino acid transporter, which can transport all kinds of amino acids but does not participate in amino acid catabolism. Moreover, butyric acid can inhibit the proliferation of intestinal pathogenic microorganisms and harmful bacteria and promote the colonization and development of beneficial bacteria such as *Bifidobacteria*, *Lactobacillales* and *Clostridiales*, and *Bifidobacteriales* can produce conjugated linoleic acid in the metabolic process [25]. Conjugated linoleic acid can improve the content of immunoglobulin in animal serum and the activity of antioxidant enzymes in the liver [26,27]. Therefore, *Lactobacillales*, *Clostridiales* and *Bifidobacteriales* play an important role in the immune process of animals. In addition to producing lactic acid and acetic acid [28], *Bifidobacteriales* can also promote the production of antibodies and cytokines, thereby enhancing the disease resistance of animal bodies [29]. Liu et al. [30] also showed that *Lactoales* acid bacteria can improve the intestinal health of piglets. Therefore, partially replacing corn with barley and wheat can effectively improve intestinal health by increasing the relative abundance of beneficial bacteria in growing pigs, enabling barley and wheat to achieve the same growth performance as corn when fed to growing pigs. This study is significant in reducing the waste of feed resources and saving production costs.

## 5. Conclusions

Wheat and barley partially replacing corn had no significant effect on growing pigs’ growth performance, blood antioxidant capacity and nutrient digestibility. However, the villus height in the duodenum of growing pigs was increased in the wheat group. The contents of volatile fatty acids in the cecum and colonic digesta of growing pigs in the barley and wheat groups increased. In addition, the relative abundance of beneficial bacteria was increased and the relative abundance of harmful bacteria was decreased in the barley and wheat groups. In conclusion, under the condition of ensuring an appropriate protein and amino acid balance in growing pigs, barley and wheat partially replacing corn energy as feedstuffs does not affect the growth performance of pigs but can regulate intestinal flora and promote intestinal health.

## Figures and Tables

**Table 1:** Ingredient composition and nutrient levels of the experimental diets (%, as-fed basis).

Items	Treatments		
	CON	WH	BL
Ingredients			
Corn	66.00	35.00	33.00
Wheat	0	38.00	0
Barley	0	0	37.25
Soybean meal	20.35	14.00	16.00
Wheat bran	8.00	7.45	6.39
Soybean oil	0.3	0.20	2
L-lysine-HCl	0.25	0.34	0.32
DL-methionine	0	0	0.06
L-tryptophan	0	0	0
L-threonine	0.1	0.06	0.09
L-valine	0	0	0.02
Dicalcium phosphate	1.70	1.70	1.70
Limestone	0.35	0.30	0.20
Choline chloride	0.35	0.35	0.35
Salt	0.35	0.35	0.37
Cr_2_O_3_	0.25	0.25	0.25
Vitamin-mineral premixa ^x^	2.00	2.00	2.00
Total	100.00	100.00	100.00
Calculated composition			
Digestible energy (MJ/kg)	14.13	14.13	14.13
Digestible lysine	0.86	0.85	0.85
Digestible methionine + cysteine	0.48	0.48	0.48
Digestible threonine	0.53	0.52	0.52
Digestible tryptophan	0.15	0.15	0.15
Digestible L-valine	0.56	0.55	0.55
Analyzed composition			
Crude protein	15.10	15.15	15.10
Calcium	0.56	0.56	0.56
Available phosphorus	0.43	0.42	0.43

^x^ Vitamin-mineral premix provided the following per kilogram of diets: vitamin A, 5512 IU; vitamin D_3_, 2200 IU; vitamin E, 30 IU; vitamin K_3_, 2.2 mg; vitamin B_12_, 27.6 μg; riboflavin, 4 mg; pantothenic acid, 14 mg; niacin, 30 mg; folic acid, 0.7 mg; thiamin, 1.5 mg; pyridoxine, 3 mg; biotin, 44 μg. Fe(FeSO_4_∙H_2_O) 50 mg; Mn(MnSO_4_) 2 mg; Zn(ZnSO_4_) 50 mg; Cu(CuSO_4_∙5H_2_O) 3.5 mg; I(KI) 0.14 mg; Se(Na_2_SeO_3_) 0.15 mg.

**Table 2:** Effects of the partial substitution of corn by wheat or barley on the growth performance of growing pigs.

Items	Treatments			SEM ^1^	*p*-Value
	CON	WH	BL		
Initial weight (kg)	51.06	50.84	50.93	0.29	0.88
Final weight (kg)	78.05	77.83	77.26	0.31	0.22
ADFI (g)	2687.01	2679.17	2656.37	22.38	0.62
ADG (g)	964.29	963.57	940.48	7.97	0.10
F/G	2.79	2.78	2.82	0.04	0.69

^1^ SEM, standard error of the mean (n = 6). ADFI, Average daily feed intake, ADG, Average daily gain; F/G, Feed over gain ratio. CON, basal diet group; WH, wheat diet group; BL, barley diet group.

**Table 3:** Effects of the partial substitution of corn by wheat or barley on the blood antioxidant capacity of growing pigs.

Items	Treatments			SEM ^1^	*p*-Value
	CON	WH	BL		
SOD (IU/mL)	124.44	124.78	125.77	3.61	0.96
MDA (nmol/mL)	1.69	1.73	1.66	0.08	0.81
GSH-Px (IU/mL)	688.52	678.46	680.18	11.42	0.72
T-AOC (IU/mL)	2.57	2.54	2.52	0.08	0.89

^1^ SEM, standard error of the mean (n = 6). SOD, superoxide dismutase; MDA, malondialdehyde; GSH-Px, glutathione peroxidase; T-AOC, total antioxidant capacity. CON, basal diet group; WH, wheat diet group; BL, barley diet group.

**Table 4:** Effects of partial substitution of corn by wheat or barley on apparent digestibility of total Intestinal Nutrients in growing pigs (%).

Items	Treatments			SEM ^1^	*p*-Value
	CON	WH	BL		
Dry matter	81.17	80.58	80.36	0.27	0.13
Organic matter	83.29	82.72	82.54	0.29	0.19
Crude protein	75.19	76.31	76.22	0.57	0.33
Gross energy	81.28	80.39	80.46	0.35	0.17

^1^ SEM, standard error of the mean (n = 6). CON, basal diet group; WH, wheat diet group; BL, barley diet group.

**Table 5:** Effects of the partial substitution of corn by wheat or barley on the villus height and crypt depth of growing pigs (µm).

Items	Treatments			SEM ^1^	*p*-Value
	CON	WH	BL		
Duodenum					
Villus height	472.13 ^b^	535.06 ^a^	478.34 ^b^	14.73	0.02
Crypt depth	314.53	303.27	321.01	7.76	0.31
Villus height/Crypt depth	1.50 ^b^	1.76 ^a^	1.49 ^b^	0.05	0.01
Jejunum					
Villus height	505.47	496.86	483.62	10.91	0.39
Crypt depth	325.15	340.22	344.41	20.04	0.78
Villus height/Crypt depth	1.58	1.48	1.42	0.08	0.44
Ileum					
Villus height	497.68	481.12	510.48	15.91	0.45
Crypt depth	308.71	348.05	332.66	14.95	0.22
Villus height/Crypt depth	1.64	1.39	1.55	0.11	0.32

^1^ SEM, standard error of the mean (n = 6). ^a^^, b^ Different superscripts within a row indicate a significant difference (*p* < 0.05). CON, basal diet group; WH, wheat diet group; BL, barley diet group.

**Table 6:** Effects of the partial substitution of corn by wheat or barley on volatile fatty acids in the cecum and colon of growing pigs (mg/kg).

Items	Treatments			SEM ^1^	*p*-Value
	CON	WH	BL		
Cecum					
Acetic acid	4956.20 ^b^	5180.10 ^b^	6214.76 ^a^	274.98	0.02
Propionic acid	2360.80 ^b^	2595.71 ^ab^	3088.21 ^a^	161.17	0.02
Isobutyric acid	64.72	103.71	85.06	13.03	0.18
Butyric acid	987.60 ^b^	1458.21 ^a^	1659.04 ^a^	167.98	0.03
Isovaleric acid	83.77	109.48	110.47	14.40	0.40
Valeric acid	165.25	232.45	238.88	21.78	0.10
Total VFA	8618.34 ^b^	9679.66 ^b^	11,396.42 ^a^	560.65	0.02
Colon					
Acetic acid	4220.56	4178.09	4633.12	134.98	0.08
Propionic acid	2444.48 ^b^	2156.67 ^b^	2885.20 ^a^	168.99	0.03
Isobutyric acid	48.58	36.15	55.44	5.01	0.08
Butyric acid	1164.78	1091.45	1355.02	99.94	0.27
Isovaleric acid	91.87	80.82	97.83	4.89	0.10
Valeric acid	196.56	193.67	206.67	15.01	0.77
Total VFA ^2^	8166.82 ^b^	7736.85 ^b^	9233.28 ^a^	280.89	0.01

^1^ SEM, standard error of the mean (n = 6). ^2^ Total VFAs = Acetate + Propionate + Butyrate + Valerate + Isobutyrate + Isovalerate. ^a, b^ Different superscripts within a row indicate a significant difference (*p* < 0.05). CON, basal diet group; WH, wheat diet group; BL, barley diet group.

## Data Availability

All of the data is contained within the article.

## Abbreviations

WH: wheat; BL, barley; CON, control; ADG, average daily gain; ADFI, average daily feed intake; F/G, feed to gain ratio; SOD, superoxide dismutase; MDA, malondialdehyde; GSH-Px, glutathione peroxidase; T-AOC, total antioxidant capacity; SCFA, Short chain fatty acid; ATTD, apparent total tract digestibility; VFA, volatile fatty acid; BW, body weight; CP, crude protein; Cr, chromium; DM, dry matter; GE, gross energy; OM, organic matter; OTU, operational taxonomic unit; PCoA, principal coordinate analysis; LEfSe, linear discriminant analysis effect size; LDA, latent dirichlet allocation; PCA, principal component analysis.
